# Supplementary material for: Safety, Immunogenicity, and Efficacy of COVID-19 Vaccines in Children and Adolescents: A Systematic Review
Source: Vaccines (Basel). 2021 Sep 29;9(10):1102. doi: 10.3390/vaccines9101102 (PMC8539812; doi:10.3390/vaccines9101102)
Supplement: Supplementary file 1 [file vaccines-09-01102-s001.zip › supplementary table S2.pdf]

Supplementary Table S2. Detailed Search Strategy

**PubMed（N=1033）**
#1 "COVID-19"[Mesh]
#2 "SARS-CoV-2"[Mesh]
#3 "COVID-19"[Title/Abstract]
#4 "SARS-COV-2"[Title/Abstract]
#5 "SARS COV 2"[Title/Abstract]
#6 "Novel coronavirus"[Title/Abstract]
#7 "2019-novel coronavirus"[Title/Abstract]
#8 "Coronavirus disease-19"[Title/Abstract]
#9 "Coronavirus disease 19"[Title/Abstract]
#10 "Coronavirus disease 2019"[Title/Abstract]
#11 "COVID 19"[Title/Abstract]
#12 "Novel CoV"[Title/Abstract]
#13 "2019-nCoV"[Title/Abstract]
#14 "2019 nCoV"[Title/Abstract]
#15 "2019-CoV"[Title/Abstract]
#16 OR/#1-#15
#17 "vaccin*"[Title/Abstract]
#18 "Vaccines"[Mesh]
#19 #17 OR #18
#20 #16 AND #19
#21 "COVID-19 Vaccines"[Mesh]
#22 #20 OR #21
#23 "Adolescent"[Mesh]
#24 "Adolescen*"[Title/Abstract]
#25 "Teen*"[Title/Abstract]
#26 "Youth*"[Title/Abstract]
#27 "juvenile*"[Title/Abstract]
#28 "puberty"[Title/Abstract]
#29 "young*"[Title/Abstract]
#30 "Child" [Mesh]
#31 "child*" [Title/Abstract]
#32 "Pediatrics" [Mesh]
**Web of Science（N=913）**
#1 "SARS-CoV-2"[Topic]
#2 "Novel coronavirus"[Topic]
#3 "2019-novel coronavirus"[Topic]
#4 "Coronavirus disease 19"[Topic]
#5 "Coronavirus disease 2019"[Topic]
#6 "COVID-19"[Topic]
#7 "Novel CoV"[Topic]
#8 "2019-nCoV"[Topic]

#9 OR/#1-#8<br>#10 Adolescen* (Topic)<br>#11 young* (Topic)<br>#12 Pediatrics (Topic)<br>#13 Child* (Topic)<br>#14 Infant (Topic)<br>#15 newborn* (Topic)<br>#16 neonat* (Topic)<br>#17 Youth* (Topic)<br>#18 student* (Topic)<br>#19 OR/#10-#18<br>#20 vaccin* (Topic)<br>#21 #9 AND #19 AND 20<br>#22 #21 AND limit Database: Pediatrics (Research Areas)
**WHO（N=1006）**<br>#1 tw:(adolescen*)<br>#2 tw:(teen*)<br>#3 tw:(youth*)<br>#4 tw:(juvenile*)<br>#5 tw:(puberty)<br>#6 tw:(young*)<br>#7 tw:(child*)<br>#8 tw:(pediatric*)<br>#9 tw:(paediatric*)<br>#10 tw:(infant*)<br>#11 tw:(neonat*)<br>#12 tw:(newborn*)<br>#13 tw:(baby)<br>#14 tw:(trottie*)<br>#15 tw:(babies)<br>#16 tw:(kids)<br>#17 tw:(toddler*)<br>#18 tw:(pre-school*)<br>#19 tw:(preschool*)<br>#20 tw:(kindergarten*)<br>#21 tw:(kinder-garten*)<br>#22 tw:(girl*)<br>#23 tw:(boy*)<br>#24 tw:(student*)<br>#25 tw:(junior*)<br>#26 tw:(pubescent)<br>#27 OR/#1-#26<br>#28 tw:(vaccin**)<br>#29 #27 AND #28

#30 #29 AND la:("en")
**CNKI（N=76）**<br>#1 '儿童' (TOPIC)<br>#2 '幼儿' (TOPIC)<br>#3 '婴儿' (TOPIC)<br>#4 '新生儿' (TOPIC)<br>#5 '青少年' (TOPIC)<br>#6 '小儿' (TOPIC)<br>#7 OR/#1-#6<br>#8 '新型冠状病毒' (TOPIC)<br>#9 'COVID-19' (TOPIC)<br>#10 'COVID 19' (TOPIC)<br>#11 '2019-nCoV' (TOPIC)<br>#12 '2019 nCoV' (TOPIC)<br>#13 '2019-CoV' (TOPIC)<br>#14 '2019 CoV' (TOPIC)<br>#15 'SARS-CoV-2' (TOPIC)<br>#16 'SARS COV 2' (TOPIC)<br>#17 '新冠肺炎' (TOPIC)<br>#18 OR/#8-#17<br>#19 '疫苗' (TOPIC)<br>#20 '接种' (TOPIC)<br>#21 '免疫' (TOPIC)<br>#22 OR/#19-#21<br>#23 #1 AND #7 AND #18
