# Supplementary material for: Safety, Immunogenicity, and Efficacy of COVID-19 Vaccines in Children and Adolescents: A Systematic Review
Source: Vaccines (Basel). 2021 Sep 29;9(10):1102. doi: 10.3390/vaccines9101102 (PMC8539812; doi:10.3390/vaccines9101102)
Supplement: Supplementary file 1 [file vaccines-09-01102-s001.zip › supplementary table S3.pdf]

Supplementary Table S3 Ongoing clinical trials on COVID-19 vaccination in children and adolescents

Trial ID	Registration date	Recruitment stage	Country	Participants' age	Target size	Study type	Intervention	Primary outcome
RPCEC00000374	10/06/2021	Not Recruiting	Cuba	3-18	350	Interventional Phase 1/2	FINLAY-FR-2+FINLAY-FR-1A	Phase I: SAEs (It will measure as: -Occurrence of the SAE (Yes, No), - Duration (Time from start date until end date of event), -Description of the event, Result (Recovered, Recovered with squeals, Persists, Death, Unknown), - Causality (Causal association consistent with vaccination, Undetermined, Inconsistent causal association with vaccination, not classifiable). Measurement time: daily for 28 days after each dose.<br>Phase II: Concentration of specific anti-RBD IgG antibodies (Percentage of subjects with seroconversion 4-fold to pre-vaccination). Measurement time: Day 0, 42 and 70
NCT04918797	18/05/2021	Recruiting	India	2-18	525	Interventional Phase 2/3	COVAXIN	Reactogenicity; Immunogenicity
NCT04917523	04/06/2021	Not recruiting	United Arab Emirates	⩾3	1800	Interventional Phase 3	SARS-CoV-2 Vaccine (Vero Cell), Inactivated	The four-fold increase rate of anti-SARS-CoV-2 neutralizing antibody; GMT of anti-SARS-CoV-2 neutralizing antibody
NCT04916886	30/05/2021	Recruiting	China	6-59	2016	Interventional N/A	Recombinant Novel Coronavirus Vaccine (Adenovirus Type 5 Vector)	GMT of anti-SARS-CoV-2 specific neutralizing antibody
NCT04895982	05/05/2021	Not recruiting	United States, Brazil, Germany	⩾2	360	Interventional Phase 2	BNT162b2	Percentage of participants reporting local reactions; Percentage of participants reporting systemic events; Percentage of participants reporting AEs; Percentage of participants reporting SAEs; GMTs of all participants, measured by SARS-CoV-2 neutralising titers, without serological or virological evidence of past SARS-CoV-2 infection and

								with an immunocompromised state, as specified in the protocol; Percentage of participants reporting AEs
NCT04894448	18/05/2021	Recruiting	Canada	⩾16	500	Observational	COVID-19 Vaccine	immunogenicity of COVID-19 vaccination
NCT04884685	07/05/2021	Not recruiting	China	3-17	500	Interventional Phase 2	SARS-CoV-2 Inactivated Vaccine;	Safety index-incidence of ARs
NCT04869592	24/04/2021	Recruiting	China	⩾3	3580	Interventional Phase 1/2	Recombinant SARS-CoV-2 Vaccine (CHO cell)	the incidence and severity of any ARs/AEs within 30 minutes after each dose of vaccination; the incidence of abnormal blood biochemistry, blood routine, blood coagulation function and urine routine on the 4th day after each dose of vaccination; the incidence and severity of ARs/AEs within 0-7 days after each dose of vaccination; the incidence and severity of non-collective ARs/AEs within 30 days after each dose of vaccination; the incidence and severity of AEs leading to withdrawal within 30 days after each dose of vaccination; the incidence of SAE from the first dose of vaccination to 12 months after the full course of vaccination; the incidence of AESI from the first dose of vaccination to 12 months after the full course of vaccination; GMT of SARS-CoV-2 specific neutralizing antibody (wild Strains) on the 15th day after the full course of vaccination; the incidence and severity of any ARs/AEs within 30 minutes after each dose of vaccination; the incidence and severity of ARs/AEs within 0-7 days after each dose of vaccination; the incidence and severity of non-collective ARs/AEs within 30 days after each dose of vaccination; the incidence and severity of non-collective ARs/AEs within 40 days after each dose of vaccination; the incidence and severity of non-collective ARs/AEs within 60 days after each dose of vaccination; the incidence and severity of AEs leading to withdrawal within 30 days

								after each dose of vaccination; the incidence and severity of AEs leading to withdrawal within 40 days after each dose of vaccination; the incidence and severity of AEs leading to withdrawal within 60 days after each dose of vaccination; the incidence of SAE from the first dose of vaccination to 12 months after the full course of vaccination; the incidence of AESI from the the first dose of vaccination to 12 months after the full course of vaccination; GMT of SARS-CoV-2 specific neutralizing antibody (wild Strains) on the 15th day after the full course of vaccination
NCT04865900	28/04/2021	Recruiting	Israel	16-60	500	Observational	Diagnostic Test: Laboratory Blood Tests; Diagnostic Test: ECG; Diagnostic Test: PCR; Diagnostic Test: Antibody test; Diagnostic Test: Echocardiography; Diagnostic Test: MRI	Elevated Troponin
NCT04863638	27/04/2021	Recruiting	China	⩾3	4400	Interventional Phase 4	COVID-19 Vaccine (Vero Cell), Inactivated;	GMT of anti-SARS-CoV-2 neutralizing antibody; The four-fold increase rate of anti-SARS-CoV-2 neutralizing antibody
NCT04848584	30/03/2021	Not recruiting	NR	⩾16	999	Observational	Pfizer-BioNTech COVID-19 Vaccine	The effectiveness of 2 doses of BNT162b2 (i.e., fully vaccinated) against hospitalization for ARI due to SARS-CoV-2 infection
NCT04832932	02/04/2021	Recruiting	United States; Kenya; United Kingdom;	⩾16	1000	Observational	COVID-19 vaccine	ARs/AEs
NCT04816643	19/03/2021	Recruiting	United States; Finland;	6m-11y	4644	Interventional Phase 1/2	BNT162b2	Percentage of participants in Phase 1 reporting local reaction in each dose level in each age group; Percentage of participants in Phase 1

			Poland; Spain					reporting systemic events in each dose level in each age group; Percentage of participants in Phase 1 reporting AEs in each dose level in each age group; Percentage of participants in Phase 1 reporting SAEs in each dose level in each age group; Percentage of participants in Phase 2/3 reporting local reaction in each dose level in each age group; Percentage of participants in Phase 2/3 reporting systemic events in each dose level in each age group; Percentage of participants in Phase 2/3 reporting AEs in each dose level in each age group; Percentage of participants in Phase 2/3 reporting SAEs in each dose level in each age group; In Phase 2/3 participants, GMR of SARS-CoV-2 neutralizing titers in participants in each age group at selected dose level to those 16 to 25 years of age in study C4591001
NCT04811391	19/03/2021	Recruiting	Albania	⩾16	1500	Observational	COVID-19 vaccine	COVID-19 vaccine effectiveness; COVID-19 PCR confirmation
NCT04800133	01/03/2021	Recruiting	China	11-100	900	Interventional Phase 2	Tozinameran; Oxford-AstraZeneca COVID-19 vaccine; CoronaVac	ARs/AEs; Binding antibody response on ELISA; Neutralizing antibody response on PRNA; T cell response on flow cytometry
NCT04796896	11/03/2021	Recruiting	United States	6m-11y	6750	Interventional Phase 2/3	mRNA-1273	Number of Participants with Solicited Local and Systemic ARs; Number of Participants with Unsolicited AEs; Number of Participants with MAAEs; Number of Participants with SAEs; Number of Participants with AEs of Special Interest, including Multisystem Inflammatory Syndrome in Children (MIS-C);Number of Participants with Serum Antibody (Ab) Levels that Meet or Exceed the Threshold of Protection from COVID-19; GM of SARS-CoV-2 Specific Neutralizing Antibody (nAb); Seroresponse Rate of Vaccine Recipients
NCT04773067	23/02/2021	Recruiting	China	12-85	3850	Interventional	UB-612	GMT of SARS-CoV-2 neutralizing antibody; Seroconversion rate

			(Taiwan)			Phase 2		(SCR) of SARS-CoV-2 neutralizing antibody; Safety evaluation
NCT04761822	17/02/2021	Recruiting	United States	12-69	3400	Interventional Phase 2	Moderna COVID-19 Vaccine; Pfizer-BioNTech COVID-19 Vaccine	Proportion of participants who experience a systemic allergic reaction to either dose of the Pfizer-BioNTech COVID-19 Vaccine; Proportion of participants who experience a systemic allergic reaction to either dose of the Moderna COVID-19 Vaccine
NCT04713553	15/01/2021	Recruiting	United States	12-50	1530	Interventional Phase 3	BNT162b2	GMR of SARS-CoV-2 full-length S-binding antibody levels between US lots (Arms 1, 2 and 3) in participants without evidence of infection during the study; GMR of SARS-CoV-2 full-length S-binding antibody levels between the EU lot (Arm 4) and pooled US lots (Arms 1, 2, and 3) in participants without evidence of infection during the study; GMR of SARS-CoV-2 neutralizing antibody levels between the 20-microgram dose group (Arm 5) and the corresponding 30-microgram dose group (Arm 1, 2, or 3) in participants without evidence of SARS-C0V-2 infection during the study.; Percentage of participants reporting local reactions; Percentage of participants reporting systemic events; Percentage of participants reporting AEs; Percentage of participants reporting SAEs; GMT of SARS-CoV-2 reference strain and B.1.351 strain neutralizing antibody levels for Booster Arm 1 (BNT162b2) and Booster Arm 2 (BNT162b2.B.1.351).;Geometric Mean IgG Concentrations (GMC) of SARS-CoV-2 full-length S-binding antibody levels for Booster Arm 1 (BNT162b2) and Booster Arm 2 (BNT162b2.B.1.351).; GMFR of SARS-CoV-2 reference strain and B.1.351 strain neutralizing antibody levels for Booster Arm 1 (BNT162b2) and Booster Arm 2 (BNT162b2.B.1.351).; GMFR of SARS-CoV-2 full-length S-binding antibody levels for Booster Arm 1 (BNT162b2) and Booster Arm 2

								(BNT162b2.B.1.351).;Percentages of participants with seroresponse (based on neutralizing titers) to the reference strain.; Percentages of participants with seroresponse (based on neutralizing titers) to the B.1.351 strain.
NCT04649151	30/11/2020	Not recruiting	United States	12-17	3732	Interventional Phase 2/3	mRNA-1273	Number of Participants with Solicited Local and Systemic ARs; Number of Participants with Unsolicited AEs; Number of Participants with SAEs, MAAEs, or AEs of Special Interest of Multisystem Inflammatory Syndrome in Children (MIS-C); Number of Participants With Serum Antibody (Ab) Levels that Meet or Exceed the Threshold of Protection From COVID-19; GM of the Serum Ab Level; Seroresponse Rate of Vaccine Recipients
NCT04611802	30/10/2020	Recruiting	United States; Mexico; Puerto Rico;	⩾12	33000	Interventional Phase 3	SARS-CoV-2 rS/Matrix-M1 Adjuvant	Pediatric Expansion: Incidence and Severity of SAEs from Month 12 to Month 24;Pediatric Expansion: Incidence and Severity of MAAEs Attributed to Study Vaccine from Month 12 to Month 24;Pediatric Expansion: Incidence and Severity of AESIs from Month 12 to Month 24;Pediatric Expansion: Antibodies to SARS-CoV-2 Nucleoprotein (NP) at Specific Time Points; Pediatric Expansion: Deaths Due to Any Cause; Main Study and Pediatric Expansion: Participants with Symptomatic Mild, Moderate, or Severe Coronavirus Disease 2019 (COVID-19);Pediatric Expansion: Reactogenicity Incidence and Severity; Pediatric Expansion: Incidence and Severity of MAAEs Through Day 49;Pediatric Expansion: Incidence and Severity of Unsolicited AEs Through Day 49;Pediatric Expansion: Incidence and Severity of MAAEs Attributed to Study Vaccine Through Month 12;Pediatric Expansion: Incidence and Severity of SAEs Through Month 12;Pediatric Expansion: Incidence and Severity of AEs of

								Special Interest Through Month 12
NCT04566770	22/09/2020	Recruiting	China	⩾6	481	Interventional Phase 2	Recombinant Novel Coronavirus Vaccine (Adenovirus Type 5 Vector);	Safety indexes of ARs; Immunogenicity indexes of GMT; Immunogenicity indexes of neutralizing antibody
NCT04551547	15/09/2020	Recruiting	China	3-17	552	Interventional Phase 1/2	inactivated SARS-CoV-2 vaccine a	Safety index-incidence of ARs; Immunogenicity index-seroconversion rates of neutralizing antibody
NCT04528719	20/08/2020	Recruiting	United States	12m-79y	620	Interventional Phase 1	mRNA-1345	Number of Participants with Solicited Local and Systemic ARs; Number of Participants with Unsolicited AEs; Number of Participants with Serious AEs or MAAEs
NCT04471519	11/07/2020	Not recruiting	India	12-65	755	Interventional Phase 1/2	BBV152	Frequency of Grade 1 or higher solicited AEs; Frequency of Grade 2 or higher lower respiratory infections (LRI); Frequency of SAEs; Percentage of vaccinees with a =4-fold rise in serum RSV-neutralizing antibody titer; Peak titer of vaccine virus shed; Proportion of vaccinees infected with vaccine virus in Group 1
NCT04368728	27/04/2020	Recruiting	United States; Argentina; Brazil; Germany; South Africa; Turkey	⩾12	43998	Interventional Phase 2/3	BNT162b1; BNT162b2; BNT162b2SA	Phase 1: Occurrence of AEs and SAEs<br>Phase 2: Evaluation of Neutralizing Antibody Titers
NCT04299724	05/03/2020	Recruiting	China	6m-80y	100	Interventional Phase 1	Pathogen-specific aAPC	Percentage of participants in Phase 1 reporting local reactions; Percentage of participants in Phase 1 reporting systemic events; Percentage of participants in Phase 1 reporting AEs; Percentage of participants in Phase 1 reporting SAEs; Percentage of Phase 1 participants with abnormal hematology and chemistry laboratory values; Percentage of Phase 1 participants with abnormal hematology

								and chemistry laboratory values; Percentage of Phase 1 participants with abnormal hematology and chemistry laboratory values; Percentage of Phase 1 participants with grading shifts in hematology and chemistry laboratory assessments; Percentage of Phase 1 participants with grading shifts in hematology and chemistry laboratory assessments; Percentage of Phase 1 participants with grading shifts in hematology and chemistry laboratory assessments; In the first 360 participants randomized into Phase 2/3, percentage of participants reporting local reactions; In the first 360 participants randomized into Phase 2/3, percentage of participants reporting systemic events; In the first 360 participants randomized into Phase 2/3, percentage of participants reporting AEs; In the first 360 participants randomized into Phase 2/3, percentage of participants reporting SAEs; In a subset of at least 6000 participants randomized in Phase 2/3, percentage of participants reporting local reactions; In a subset of at least 6000 participants randomized in Phase 2/3, percentage of participants reporting systemic events; Percentage of participants in Phase 2/3 reporting AEs; Percentage of participants in Phase 2/3 reporting SAEs; Confirmed COVID-19 in Phase 2/3 participants without evidence of infection before vaccination; Confirmed COVID-19 in Phase 2/3 participants with and without evidence of infection before vaccination; Percentage of participants 12-15 years of age in Phase 3 reporting AEs; Percentage of participants 12-15 years of age in Phase 3 reporting AEs; In participants 12-15 years of age randomized in Phase 3, percentage of participants reporting local reactions; In participants 12-15 years of

								age randomized in Phase 3, percentage of participants reporting systemic events; In participants who receive BNT162b2SA given as 1 or 2 doses, percentage of participants reporting AEs; In participants who receive BNT162b2SA given as 1 or 2 doses, percentage of participants reporting SAEs; In participants, who receive BNT162b2SA given as 1 or 2 doses, percentage of participants reporting local reactions; In participants who receive BNT162b2SA given as 1 or 2 doses, percentage of participants reporting systemic events; In participants who receive a third dose of BNT162b2, percentage of participants reporting AEs; In participants who receive a third dose of BNT162b2, percentage of participants reporting SAEs; In participants who receive a third dose of BNT162b2, percentage of participants reporting local reactions; In participants who receive a third dose of BNT162b2, percentage of participants reporting systemic events; Noninferiority of the SARS-CoV-2 reference strain neutralizing titers after a third dose of BNT162b2 at 30 g compared to after 2 doses of BNT162b2, in the same individuals; Noninferiority of the SARS-CoV-2 SA strain neutralizing titers after one dose of BNT162b2SA compared to the SARS-CoV-2 reference strain neutralizing titers after 2 doses of BNT162b2, in the same individuals; Noninferiority of the SARS-CoV-2 SA strain neutralizing titers after 2 doses of BNT162b2SA compared to the SARS-CoV-2 reference strain neutralizing titers after 2 doses of BNT162b2
NCT04276896	17/02/2020	Recruiting	China	6m-80y	100	Interventional Phase 1/2	LV-SMENP-DC vaccine	Frequency of vaccine events; Frequency of serious vaccine events; Proportion of subjects with positive T cell response

GMT: The Geometric Mean Titer; AEs: Adverse Events; SAEs: Serious Adverse Events; MAAEs: Medically Attended Adverse Events; Ars: adverse reactions; GMFR: Geometric Mean Fold

Rise; GM: Geometric Mean; GMR: Geometric Mean Ratio; N/A: Not Applicable
